# Global Health Determinants Perceived and Expressed by Children and Adolescents Between 6 and 17 Years: A Systematic Review of Qualitative Studies

**DOI:** 10.3389/fped.2020.00115

**Published:** 2020-04-03

**Authors:** Maéliane Deyra, Chloé Gay, Laurent Gerbaud, Pauline Berland, Frank Pizon

**Affiliations:** ^1^University of Clermont Auvergne, CNRS, SIGMA Clermont, Institut Pascal, Clermont-Ferrand, France; ^2^University of Clermont Auvergne, CHU, CNRS, SIGMA Clermont, Institut Pascal, Clermont-Ferrand, France

**Keywords:** conceptions, children, health determinants, qualitative studies, systematic review

## Abstract

**Aim:** To identify the determinants of global health in the literature as perceived and expressed by children and adolescents in order to adapt prevention actions to this young audience. To also question the pertinence of a qualitative approach when interviewing children.

**Method:**Systematic review of the literature from PubMed, Google Scholar, CINAHL, PsycINFO databases. The studies selected used qualitative methods alone for investigating the views of health determinants in children and adolescents.

**Results:**185 articles were read to reach a final selection of 13 articles on global health, excluding studies with children who were ill, studies using quantitative, mixed, or retrospective methodologies, and those dealing exclusively with themes of health. Collecting information from children and adolescents showed the pertinence and effectiveness of qualitative methods. It also appears necessary to explore new paths: improving and adapting the tools and methodological supports used and combining them to enrich repositories.

**Conclusion:**The small amount of qualitative data available with the views of children and adolescents on health determinants requires that new studies with better adapted collection methodologies be set up. To increase pertinence and effectiveness among a young audience, it is necessary, considering the methodologies identified during this literature review, to turn toward a multi-phase method that combines these methods. A methodology in several phases allows each one to use a different approach with young people and to obtain richer and more varied information. A corpus of images appeared as a powerful tool for collection: it facilitates children's capacity for oral expression and places the researcher in a position of listening.

## Introduction

Public health has seen a notable evolution over time, moving toward a much more global approach that integrates influencing factors such as social, economic, and ecological dimensions of health (1). The current holistic approach to health positions health as a relationship between an individual, their own body, and the environment in which they live. Global health is therefore not confined to identifying infectious and functional causes in pathological cases, but also highlights the environmental, psychosocial, and socioeconomic reasons that create the context of social inequalities in health (2).

The issue of social inequalities in health is increasingly shared today (3). This is echoed in current public health policies (4). The latter are based on the observation that in terms of access to healthcare, social and territorial inequalities feed a system that, overall, is not adapted to the evolution of needs (the multiplication of chronic diseases). Current public health policies are experiencing an epistemological rupture. They are not centered on risk factors and protection factors but focus instead on health determinants.

Health determinants correspond to socioeconomic factors that act interdependently with the environment, but also with individual behavior, characterizing a health status through these complex interactions. According to the Edmond et al. report of 2020, these determinants therefore do not act in isolation: it is the combination of their effects that influences a health status (5). There are different explanatory models of these health determinants. Some emphasize the role of birth conditions and early childhood life that, if unfavorable, lay the foundations for creating inequality. Others lean on the cumulative effect of unfavorable social and economic determinants that combine and interfere with each other throughout the course of a life. The example of the Dahlgren and Whitehead model (6) presents health determinants on four levels (7): the “factors linked to personal lifestyle,” the “social and community networks,” the “factors linked to living and work conditions,” and the “socioeconomic, cultural, and environmental conditions.” These levels are not independent from each other, they interact (8). Health determinants also provide a more correct, more complete, and more nuanced image of reality (9).

Their inclusion in prevention programs has been shown to be an efficiency factor for interventions (10–12).

Public health policy strategies now prioritize prevention over the curative, and action over health determinants (9). This implicitly requires an exhaustive knowledge of the latter. To better act, it is therefore precisely necessary to evaluate the population's level of knowledge of these health determinants, and as soon as possible. Notably, what of the children? What ideas do they have on health and its determinants?

Quantitative collection methodologies are highly defined (epistemology, quantitative sociology, sociometry). They serve to collect raw, concrete, and structured data that help to define the general conclusions of a study. They can measure the subject but they are not suited to describing it. In contrast, qualitative methods allow the subject to be given more depth in order to obtain more detailed and thoroughly explored information. Furthermore, qualitative methodologies facilitate the collection of information from young audiences. These are open methodologies that give room for the child and adolescent to speak in a constantly changing context, including amongst others the media environment, new social practices, and the evolution of consumption. It therefore appears appropriate to carry out an analysis of the literature in order to identify how health determinants are addressed by children and adolescents, and through which methodologies.

## Aims

The main aim of this literature review is to identify the health determinants expressed and perceived by children and adolescents through qualitative methodologies.

The secondary aim is to analyze the existing qualitative methodologies and to highlight the pertinence of these approaches when dealing with children, notably on questions linked to health.

## Methods

The method used is a systematic review of the literature, carried out according to the PICO model (Population, Intervention, Comparison, Results). It is registered on Prospero, the ≪international prospective register of systematic reviews≫, under the following number: CRD42019125434. The databases consulted were PubMed, Google Scholar, CINAHL, and PsycINFO.

The start of qualitative research dates back to the 1920's (13). Anthropologists and sociologists were the first to lead studies on the phenomenon of humans in their natural environment from a holistic point of view. Since the 1950's, marketing has used data collection techniques that are specific to qualitative research, such as interviews and focus groups. It was from the 1990's that health researchers appropriated these methods.

The term MESH (MEdical Subject Headings, international thesaurus serving as inquiry base in Medline) appeared in 2003. The articles selected for this literature review therefore correspond with those published from the 1990's to today. Some of them are somewhat old but the aim was not to miss any founding articles, even though the world has evolved in 40 years.

**Figure 1:**
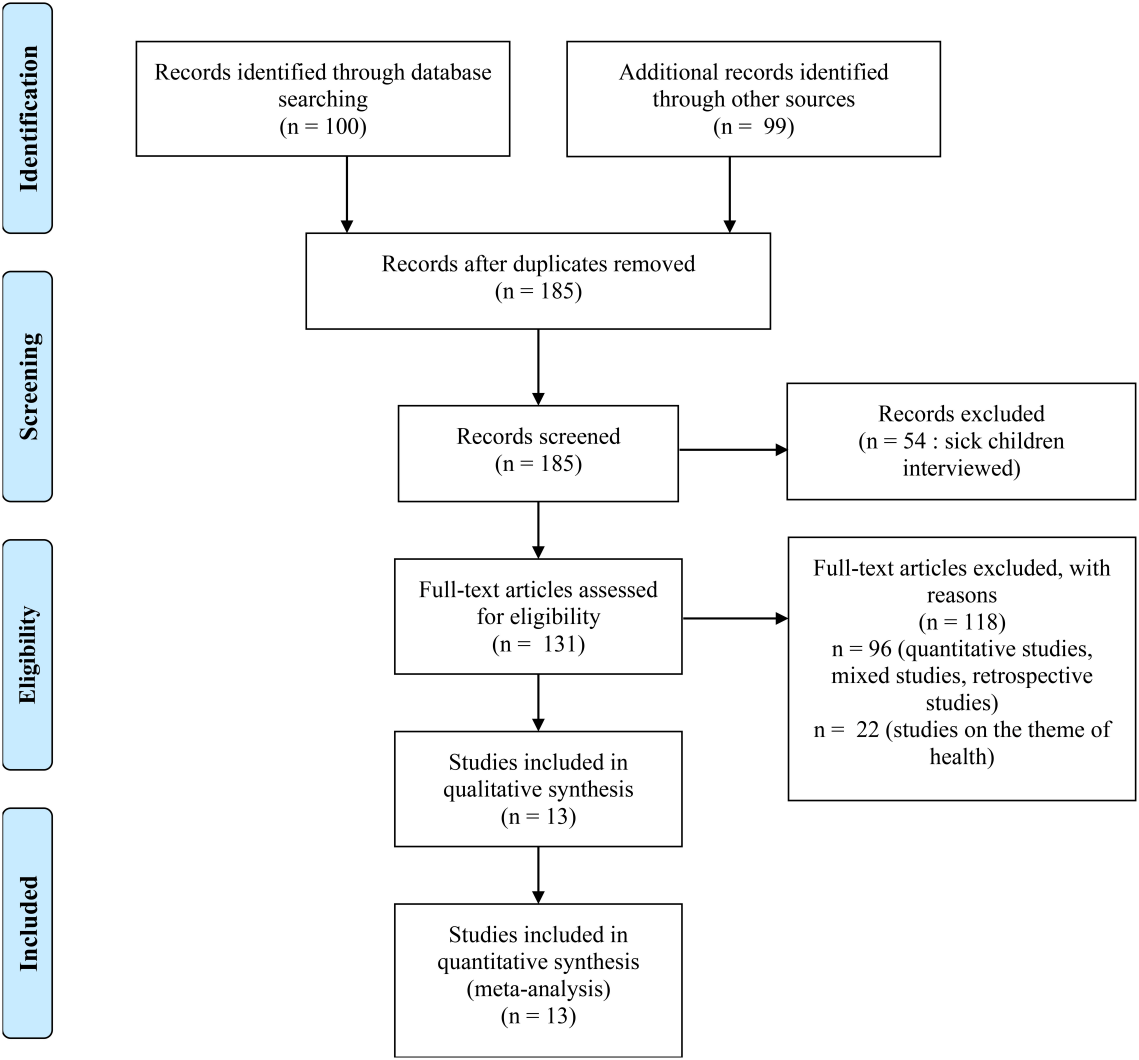
PRISMA flow diagram.

The key words used, also called “mesh” on PubMed, are the following: *child experience AND health AND qualitative study*. Only articles written in English were kept. This selection was carried out through the identification of keywords, reading titles, abstracts, and whole articles (Figure 1, prisma flow diagram). A first stage of reading allowed the elimination of a certain number of references on children who were ill. We were interested in the views on global health of healthy children. A second stage of analysis excluded references with quantitative methodologies (surveys, cohort studies) or mixed methodologies that were mainly quantitative, as well as retrospective studies. Finally, the references selected for the summary were those on global health, excluding those on themes of health.

After the removal of duplicates, the titles and abstracts of 185 articles were read. The complete reading of 131 manuscripts then allowed the selection of a smaller number of articles based on eligibility criteria, excluding studies with children who were ill and keeping those in which healthy children were questioned (*n* = 54). Only 35 studies on health or themes of health were kept. The others, of which there were 96, used a quantitative, mixed, or retrospective methodology and were excluded. This review focused on children's views of health and health determinants. As such, only the studies interviewing children through qualitative methods to collect this information were selected (*n* = 13) (Table 1) and those on specific themes of health were ultimately not taken into account (Tables 2–4).

**Table 1:** Health literature review references—Global health (*n* = 13).

**References**	**Main objective**	**Characteristics**	**Country**	**Methodology(ies)**	**Main results**
Bhagat et al. (14) [1]	To develop the theory of health literacy	8–11 years	After-school nursery United States Mid-Atlantic	Semi-structured individual interviews	Children's interpretations of the information found on nutritional labels coincide with their conceptualizations of health, their personal experiences of health
Michaelson et al. (15) [2]	“Where do young people find their ideas on health?”	10–16 years	Canada Ontario	Focus groups	Emerging categories of learning: self-reflective experience, experience of close contacts, casually observing others, and common discourse.
Piko et al. (16) [3]	Layman beliefs of young people on health	8–11 years	Two primary schools Hungary	Open questions on health/disease	Children's written answers through phrases and drawings. Knowledge: health, illness, risk of disease. Mindful of their health, positive attitude toward health and health promotion.
Priest et al. (17) [4]	Points of view of indigenous children on their health and wellbeing in an urban setting	8–12 years	Australia	Photoelicitation focus groups	Perspectives of urban indigenous children on health and wellbeing: rich understanding of the physical, socio-emotional, and cultural interconnections + the vital importance of family and community relations.
Zhang et al. (18) [5]	Health information sharing habits of Chinese adolescents on social network websites	12–17 years	Community organizations United States Chinese neighborhood in Chicago	Focus groups	Use of different social network sites. Sharing of useful and/or interesting information on their health. Problems of credibility of the health information and suggestion of evaluating the information based on personal experience, intuition, word of mouth, or online information.
Renslow et al. (19) [6]	Intercultural similarities and differences in the perception of health.	9–10 years	Public primary school United States and Guatemala.	Drawings/representative writing: good health/ bad health	Food at the heart of children's perceptions: healthy and unhealthy, + than 90% of children described food products for both subjects. Fruit: the majority of the representations of health products, followed by vegetables.
Davó-Blanes et al. (20) [7]	Suggestions of schooldchilren to promote their own health through their ideas on health.	8–12 years	Spain Alicante	Focus groups	Wide and varied notion of health. Identification of social and interpersonal determinants of health. Existing solid base for considering schoolchildren as “agents of health.”
Hernán-García et al. (21) [8]	Opinions of primary school students on the Internet as a source of advantage for health and wellbeing	6–11 years	Spain	Focus groups	Internet: tool for learning, communication, leisure, and healthcare. Children understand the influences on health and wellbeing in relation to their view of the Internet.
Onyango-Ouma et al. (22) [9]	Evolution of health/sickness concepts after a health education intervention.	10–15 years	Kenya	Interviews Drawing-writing	Children's acquisition of new concepts of health. Children can modify and expand their concepts of health/illness thanks to health education based on action. Key factors: development of appropriation through active and participative teaching and learning approaches.
Pridmore (23) [10]	“Draw and write” an innovative method for involving children in research on health.	9–10 years	Bushmen: Isolated Kalahari Ghanzi desert camp + 3 schools Botswana	Drawing and writing Staying in good health/ bad health.	What makes you healthy? Bushmen: food, exercise, medicine, and hygiene / Botswana: food. What leads to bad health? 7/11 (Bushmen) drew: alcohol and tobacco + fighting and accidents. 1 child identified an illness and another drew himself “hungry.” Drawings: 66% of the children from Botswana: sugar and sweets, 10%: children eating + water.
Bird et al. (24) [11]	Possible age differences in the perception of sickness and health by children.	5–11 years	Kindergarten/ primary schols Greece	Create two sickness/health drawings, give an explanatory title	The oldest children: more varied representations of sickness/health than the younger. Sickness: perceived as a biomedical phenomenon and health as a psychosocial phenomenon.
Eiser et al. (25) [12]	Children's ideas on health and sickness.	6–8–9–11 years	Washington United States	Interviews	Defining “being in good health” + how to stay in good health/avoid falling ill: food as a central role.
Goldman et al. (26) [13]	Conceptions of illness of young children.	4–6 years *n* = 27	New Haven United States	Individual interviews	Five characteristics of representations of illness: causality, identity, consequences, chronology, and recovery.

**Table 2:** Health literature review references—Nutritional behavior (*n* = 11).

**References**	**Main objective**	**Audience characteristics**	**Country**	**Methodology(ies)**	**Main results**
**NUTRITION**					
Fairbrother et al. (27) [1]	To explore children's understanding of family financial resources and how they perceive the link to eating healthily.	Children of 9–10 years	Two different socioeconomic schools England	Interviews with friendship groups, school debates, and individual interviews at home.	- Ideas on the interrelations between nutritional diet, cost, and health - Awareness of the influence of family financial resources on the purchase of food. -Linking of healthy eating with the consumption of fruit and vegetables
Gallegos-Martínez and Reyes-Hernández (28) [2]	To determine the social representations transmitted by caregivers, teachers, and children to food, health, and nutrition	Mothers, grandmothers, 1 father, Children of 3–7 years, and teachers	San Luis Potosi Mexico	Semi-structured interviews	Knowledge of foods, health-nutrition relationship, children's eating customs and practices, and significance of the school breakfast program.
Correa et al. (29) [3]	To understand the perceptions and attitudes of adolescents of Indian origin in India and in Canada that have the potential to contribute to healthy eating behaviors.	Adolescents aged 11–18 years from different socioeconomic backgrounds	India rural and urban, Urban Canada	Group discussions	Perceptions of healthy foods that are rich in vitamins, minerals, and fibers. Advantages identified of a healthy diet: increased energy and disease prevention. Obstacles identified: peers, availability, access, and the affordable cost of unhealthy foods.
Zeinstra et al. (30) [4]	To explore the link between children's perceptions and preferences for fruit and vegetables and their cognitive development.	Children of 4–5 years/ 7–8 years/11–12 years	Primary school Netherlands Wageningen	Duo—interviews and group discussions	The most important determinants for liking or not liking: from appearance and texture attributes for 4–5 year olds to taste attributes for 11–12 year olds. The children's knowledge of basic tastes has increased. Their understanding of health improves with age.
Fairbrother et al. (31) [5]	To explore how children understand food in everyday life and how they perceive the relationship between food and health.	Children of 9–10 years	Two schools (different socio- economic context) England	Interviews and debates + individual interviews	Access and interaction of children to a range of sources of information on health linked to food. A common method used by the children in both schools to try and understand and rationalize the message “Five fruits/vegetables per day” is based on the persistent dichotomy between natural (healthy) foods and artificial (unhealthy) foods.
**OBESITY**					
Mosley et al. (32) [6]	To explore the beliefs pertaining to behaviors linked to the obesity status of adolescents and their mothers.	Adolescents 9–13 years + their mothers	Hawaii	Semi-structured individual interviews	Certainty: diets should include fresh foods and are based on principles of variety, balance, and moderation. In describing ideal body size, mothers expressed greater concern than girls for overweight and ethno-cultural beauty standards. Mothers believe that daughters should have a positive relationship with food and apply portion control strategies. Findings reveal how mothers' and daughters' beliefs may influence daily food-related practices in adolescents.
Pagnini et al. (33) [7]	To examine similarities and differences in the perception of parents, adolescents, general practitioners, and education professionals on matters of overweight and obesity in children.	Parents of preschool-aged children, parents of primary and secondary pupils, students, early childhood personnel, teachers, general practitioners	Australia	Discussion groups and individual interviews	Perception by the respondents of weight as associated to health, to social problems, and to moral judgements. All groups recognized the emotion associated with food and weight. Common social, cultural, and psychological themes show the complexity of obesity in children as a public health problem and the need for interventions and communication taking into account the perceptions in order to be pertinent and effective.
**PHYSICAL ACTIVITY, SEDENTARISM, FOOD, OBESITY**					
Ross and Francis (34) [8]	To describe the perceptions of physical activity, the context, the facilitators, and obstacles from the point of view of immigrant children of Hispanic origin.	Hispanic children aged 6–11–14 years	After-school program United States, Pennsylvania	Observation, field notes, semi-structured interviews, and a PhotoVoice activity.	Negative attitudes of children with regards to physical activity linked to physical uneasiness, to poor athletic competence, and to security problems. Perception of physical activity and games as identical activities: “pleasure” identified as the main driver of preferences for physical activity. Facilitators and obstacles to physical activity were linked to specific factors (to parents / at home, at school, in the neighborhood).
Hesketh et al. (35) [9]	To get the point of view of children and parents on the social and environmental obstacles to healthy eating, physical activity programs and prevention of childhood obesity.	Children 7–8 and 10–11 years + parents.	Three schools in Victoria, Australia	Discussion groups: activities based on photos	Nine themes: information and sensitization, contradiction between knowledge and behavior, balanced lifestyles, local environment, obstacles to a healthy lifestyle, contradictory messages, myths, the role of schools and family, calendar and content of childhood obesity prevention strategies.
Snethen and Broome (36) [10]	To identify the point of view of children on their weight, their exercise, and their state of health.	Children 8–12 years, anglophones with body mass index > or = 95% for their age and sex.	United States	Interviews	Emerging themes: intellectual disconnect, incongruence of body image, social importance and perspectives of exercise. Identification of healthy and unhealthy behaviors: food intake and physical activity. Children's knowledge of healthy eating and physical activities disconnected from real health practices.
Hidding et al. (37) [11]	To explore the determinants of sedentary behavior in children from the point of view of children and parents.	Children 11–13 years + parents	Amsterdam	Concept mapping sessions with four groups of children and two groups of parents.	The most important determinants perceived by the child and the parent were linked to the social, cultural, and physical environment.

**Table 3:** Health literature review references—addictions (*n* = 6).

**References**	**Objective(s) of the study**	**Audience characteristics**	**Country**	**Methodology(ies)**	**Main results**
Murray (38) [1]	To understand the experience of parental alcoholism from an adolescent's perspective	Adolescents	Canada	In-depth interviews	- Resilience and strength of participants - Unique character of their stories - Therapeutic benefit for the participants in telling theirstories
Milton etal. (39) [2]	To collect the stories of early smoking in young children	Pre-adolescents 9–11years	England Liverpool	Questionnaires, individual interviews, and discussion groups	Increasing rates of experimentation Stories of early smoking in children: - Curiosity - Role ofpeers
Rugkåsa etal. (40) [3]	To understand the meaning of tobacco dependence in young people.	Children and adolescents.	Economically disadvantaged regions Northern Ireland.	Interviews	Children's conceptual difference between children smoking and adults smoking. Adults: personal reasons. Children: social reasons, to belong to a group.
Camenga etal. (41) [4]	To explore the perceptions, beliefs, and social norms of adolescents with regards to these products.	Adolescents 11–17 years.		Discussion groups	Use of tobacco-flavored products and flavored electronic cigarettes facilitates approval and acceptance of peers.
Johnson etal. (42) [5]	To describe the patterns of language used by young people to describe tobacco dependence and what this means to them.	Adolescents	Canada	Individual and open interviews.	Five aspects of tobacco dependence identified: social, pleasurable, empowering, emotional, and full-fledged.
Mermelstein and Tobacco Control Network Working Group (43) [6]	To explain the gender differences on matters of youth smoking	Adolescents of 5 ethnic groups: white, Afro-American, Hispanic, Native American, Asian American/Pacific Islander	11 American states	Discussion groups	Themes: reasons to smoke and to not smoke; images of smokers and non-smokers; messages received by the youths on the topic of smoking and not smoking, and the social context of smoking. Unequal prevalence of smoking between ethnic groups and genders.

**Table 4:** Health literature review references—Other themes (*n* = 7).

**References**	**Health theme**	**Main objective**	**Audience characteristic(s)**	**Country**	**Methodology(ies)**	**Main results**
Cosma et al., (44) [1]	Mental health	To understand the increase in mental health issues among children and adolescents	6 groups of 6-8 children 11–15 years (1 group per Country)	Serbia Czech Republic Italy England Germany	Discussion groups	Perspectives on the differences between genders and the evolution of the mental wellbeing of adolescents. Perspectives on the measures that allow the resolution of these problems.
Dale et al., (45) [2]	Health injuries	To categorize the perceptions of children on the gravity of their injuries and their explanations	Pupils	Six randomly selected schools in Sweden	Focus groups	Need for medical care, long term consequences, and knowledge of the situation with risk of injury identified as important determinants in the perception of children on the gravity of an injury. Three categories emerged from the children's explanations of their injuries: “because of me,” “because of the situation,” “just inexplicable.”
Petersson et al. (46) [3]	Quality of life	To explore the experiences of children about a structured assessment of the health-related quality of life during a patient encounter.	Children 10–17 years	Sweden	Interviews	The children stated that the assessment provided them with information on their health, which motivated them to modify their lifestyle. When the results were discussed and asked, the children felt encouraged.
Hobbs et al. (47) [4]	Body image	To examine how girls interpret advertisements for slimming products in order to determine whether misleading advertising techniques are recognized.	Girls, 9–17 years	Seven geographical regions in the United States	Qualitative method in groups of three, viewing of seven advertisements for slimming products. Sharing of their interpretations of each advertisement	Common factors in girls' interpretation of weight loss advertising: the emotional reaction to texts through identification with the characters; comparing and contrasting persuasive messages with experiences lived with family members; using prior knowledge on food management and recognizing obvious false allegations such as “rapid” or “permanent” weight loss.
Ott et al. (48) [5]	Sexuality	To analyze the way in which adolescents themselves conceptualize sexual abstinence.	Adolescents of 11–17 years	Primary health clinics. India	Semi-structured exploratory interviews	Consideration of sexual abstinence as part of a continuum of normal development. Abstinent teens at one point who then move onto sexual activity when ready. The preparation is determined by individual factors (age, life events, physical, and social maturity), relationship factors (being with the “right” person or having a solid relationship), moral and religious factors. Sex considered as an important rite of passage to adulthood.
Råssjö and Kiwanuka (49) [6]	Sexuality	To describe how young people react to their social life conditions and why they marry early, teenage pregnancy, and forced sexual relations	Youths of 15–24 years	Two study sites: slums in Kampala and a village in the Wakiso district, Uganda	Discussion groups	The subjects addressed: forced sexual relations, early marriage, contraception, teenage pregnancy, and transactional sexual relations. Descriptions of the way in which young people fall victim to harmful cultural practices, to power imbalances due to inequalities based on gender and to a lack of information and competences necessary in modern life. The young perceive themselves as a resource, ready to help other less fortunate young people to acquire knowledge on these questions of health.
Senior (50) [7]	Hygiene, quality of life, environment	To plan and assess an intervention aiming to improve the experience of pupils using toilets	Pupils of 6–10 years	Primary schools Melbourne Australia	Discussion groups	All the students said to avoid using the toilets at school. 71% (girls), 65% (boys) feared the behavior of other pupils in the toilets (reason: lack of privacy due to the bad behavior of pupils). After a renovation of the toilets, the behavior of pupils improved (37% stated that they now liked the toilets) and incidents of vandalism decreased.

## Results

### Principal Health Determinants Described

The analysis of children's words shows us that they have a wide and varied notion of health (20). In this case, they oscillated between the idea of health as synonymous with the absence of illness, and a notion that also includes physical, psychological, and social factors. Children identified their health with physical aspects linked to their development such as height and physical strength, as well as to the functional capacity that enables these aspects. They considered health as the result of certain lifestyles. This notion was also influenced by the dominant biomedical model that aims to modify individual behaviors (51). Nevertheless, the principles of a holistic concept of health were also found in their rhetoric, notably when referring to emotional elements (joy, feeling of freedom or sadness) that are associated with well-being. The environment is another aspect that children included in their perspectives of health (16). In addition, pupils associated the execution of school tasks with well-being. Moreover, they identified determinants of health linked to social aspects, notably interpersonal relations (family, peer groups), and highlighted elements of the social environment (situations of social injustice). These results show that pupils have opinions, perceptions, and notions of health, particularly in a school context. They are also able to identify their health problems and those of others, and to propose solutions to resolve them. This study (20) shows that there is in fact a solid basis for educating school pupils as agents of community health, especially with regards to initiatives of health promotion within a school itself, and therefore for making it an explicit objective of health education programs.

Another study (22) shows that the opinions of children are numerous and combine both negative and positive aspects of health at the same time. They have a global holistic view, including amongst others the feeling of well-being, happiness, performance levels, the absence of pain, a description of illness, as well as problems of hygiene. Their concept of health is multidimensional and includes different aspects that have evolved with the establishment of a health education intervention. The participative nature of this intervention that emphasized individual and collective action at school and at home further sensitized pupils to concepts of hygiene and illness. Their view of health after the intervention reflects the passage from a passive to an active approach to health: before the intervention, illness was caused, according to them, by natural phenomena (such as the rain and the sun), physical objects (such as bikes and cars), and other living beings (such as snakes and leopards). Germ theory as the cause of illness was absent in many pictures and interviews. The children considered illness as something external, often linked to destiny, over which they had no possibility to act. After the intervention, the pupils themselves took the responsibility to act upon themselves, for example by wearing clean clothes, cutting their nails, improving the school playground, and preparing food in a hygienic manner. The intervention enabled the majority of children to modify their concept of illness and integrate a diagnosis for a specific disease linked to causes and visible external signs. This is linked to the content of the intervention which was based on malaria and diarrhea, during which the pupils learned their signs and symptoms. A significant number of them then constructed a system of knowledge for themselves, allowing them to link the symptoms and causes to ease the process of action and change among families and peers.

The results of another study (14) show that health literature influences the way that children generate meaning from information on matters of health. Reading and understanding visual information on health remained an essential element in the health literacy of these young children. The participants were able to read parts of nutritional labels and to understand the aim. They knew the nutrients and ingredients that were “bad” for their health and those that were “healthy.” It became clear that giving meaning to health information (for example, understanding what sugar is) was more pertinent for acting on the way in which children conceptualized health than the information itself.

Furthermore, the main source of health information for children was their parents, with school falling in second position. Their views were linked to what they stated having heard from a parent or a teacher. The participants engaged in critical analysis by linking knowledge to their own personal experiences. The children who played a more active role in their own decisions in terms of health had more complex views on matters of health, beyond what they had been told was “good” or “bad” for their health.

For many children, health constitutes a positive resource for life (16). Beyond the biomedical approach to health, they expressed a holistic vision of health that also puts an accent on balance and harmony, a balance between themselves and their environment, which can also be considered as an ecological approach to health promotion (51, 52). As children of this age usually only have minor infections, the most important aspect of their health was the absence of bacteria or symptoms such as coughing or a runny nose. In terms of the cause of illness, most of them mentioned a contagion or infection. Fewer children emphasized behaviors linked to health (example: cigarettes, unhealthy foods, drugs, and alcohol). The environmental factors responsible for illness were mentioned a few times (example: exhaust gas, atmospheric pollution, and contaminated air). Passive smoking also appeared as a form of polluted air for them.

Children's lay concepts of health promotion and disease prevention reinforce the idea that children are mindful of their health and express positive attitudes with regards to health. They highlight a healthy lifestyle as the main resource for maintaining health and preventing the appearance of illness. For example, they suggest among other things, relaxation and sleep, regular outdoor sports activities, games with friends, a healthy diet, and vitamins. They therefore remain mainly focused on individual behaviors.

Another qualitative study allowed the exploration of health perceptions in adolescents and the methods they use to obtain knowledge on matter of health (15). The results show that there are two main methods through which young people acquire ideas on health: through didactic learning and through biological learning. The first corresponds to an implicit learning through experiences that occur naturally in their daily lives. The second corresponds to a more explicit learning that happens both in formal contexts, such is in school, but also in informal contexts, such as at home. The categories of biological learning include self-reflective experience: “the process of personal examination and exploration from a serious question, triggered by an experience that creates and clarifies “what we are,” and gives way to modified conceptual perspective” (53); experience of close contacts: corresponds with the observation of loved ones, particularly teachers and family members, which seems to shape the ideas of participants on health, whether the observed behavior is positive or negative for their health; casually observing others: corresponds with the observation of those who are not close contacts, with the advantage that the participants have little information on the histories of the people observed. There are certain similarities with the second category but there are fewer details than the stories given on the subjects they know well; Common discourse: corresponds with the social norms that demonstrate the power of a set of beliefs spread throughout groups of adolescent populations on various health behaviors.

The results show that biological learning was identified several times by the participants as much more influential in forming their ideas on health than learning carried out in didactic contexts. Some participants also underlined that didactic learning was more important for shaping their ideas on health and that it should not be underestimated.

Children consider the internet as beneficial for their well-being and associate it with doing things to feel good, to interact, or to share ideas, experiences, and feelings with others (21). The internet was also described as a tool for “health care,” learning about subjects linked to health, the acquisition of healthy habits, and knowledge of medicines and diseases. The participants also mentioned the usefulness of accessing health care services with the help of online tools such as services for making appointments: “you can look for information on a medicine or something else if you don't understand it;” “when you are ill, you can research which medicines to take;” “you can book an appointment with a doctor.” Nevertheless, participants in this study nuanced their words by declaring that the internet could be harmful to health and well-being. The children and adolescents expressed the opinion that the internet can create problems due to the limitations and credibility of the information consulted, since it constitutes an unsure and dangerous environment (18).

### Principal Methodologies Used

The methodological approaches identified in the international publications selected for this literature review follow three dominant techniques, with a more or less narrative objective, and that can be associated with each other: interviews, the “draw and write” method, and focus groups. Using drawings and carrying out focus groups sessions are the most represented methods: five made use of drawings (19, 22–24) and five had focus groups (15–21). Four studies carried out interviews (14, 22, 26) and one involved photoelicitation (17). These qualitative methods emphasize the words of the youths and consequently allow for the collection of data that may not be voluminous but is rather rich.

Drawings are sometimes used alone (16, 54) but in the above studies, they are often combined with writing so that the child or adolescent can describe and explain their drawing. This process is said to be centered on the child and adapted to their age as it allows them to express themselves at their own level. One study (16) leaned on the “draw and write” method to identify the beliefs of young people on matters of health. The children received previously prepared sheets of paper with several questions: “What does health mean to you?; Do you know what makes you ill?; What do you do to stay healthy?; What do you do to avoid being ill?.” The answers could be given in written and/or as a picture.

Drawings also allow for the analysis of intercultural similarities and differences in the perception of health by children between two countries or between different groups of ages. This is the case for the United States and Guatemala in one of these studies (19) in which the children had to draw pictures representing good health and bad health and then explain them in writing. The study “Children's understanding of health and illness” (24) aimed to measure the differences in perceptions of illness and health according to age. The “draw and write” method also has a transformative role that enables the evolution of conceptions of health and illness (22). In the context of this study, children drew pictures and wrote phrases that represented, for them, what allows them to stay in good health and conversely, what can lead to poor health. Other than helping to make perceptions, beliefs, and the thoughts of children emerge, some researchers highlight the fact that this collection method was considered as innovative in the late 1990's, allowing the involvement of children in research on health (23).

On the other hand, interviews often constitute the only tool for data collection in the methodologies observed, excluding one on the evolution of concepts of health and illness after a health education intervention: in this case, interviews are linked with the “draw and write” method (22). This guarantees the spontaneity and freedom of answers from young people when faced with the sometimes-intimidating nature of a focus group.

As for “focus groups,” they are often identified as the only method of data collection. Among the 13 studies, only once were they combined with another mode of collection, photoelicitation (17), to discover the point of view of native children on their health and well-being.

As a reminder, a focus group is a group interview technique with directed expression and questioning that allows information and data to be collected on a target subject, through group discussion rather than through individualized surveys. This is the case for a particular study (20) in which the focus group collected suggestions from schoolchildren for the promotion of their own health, based on their ideas on health. The result of this form of research reflects the reality of the interaction between the attitudes of children and adolescents, and the social processes within the group. This, for example, promoted the emergence of health information sharing habits of Chinese adolescents on social network websites (18). This technique also allows the evaluation of needs, expectations, satisfaction, or a better understanding of opinions, motivations, or behaviors. This was the case for two studies: one that aimed to know “where do young people find their ideas on health?” (15), and the other that aimed to define the opinions of primary school children on the internet tool as a resource for health and well-being (21).

For the rest, there are other methodologies that are used less often. One of them has been the object of a publication. This is “photoelicitation” (17), a method that allows the expression of a point of view on a theme shown through a corpus of images, which is presented to children or put together by themselves (55). The aim of this study was to identify the perceptions of children on their health and well-being in an urban environment.

## Discussion

We note that qualitative research works on health determinants are mainly structured around and centered on adult populations (56–58) with the logical consequence of a relative scarcity of international publications using qualitative methodologies with a young audience. Only 13 articles were selected for a more exhaustive analysis (Table 1). These works look at the way in which children and adolescents aged between 6 and 17 years consider and address health. They originated from the United States, Canada, Hungary, Australia, Spain, Guatemala, Kenya, Botswana, and Greece.

Pupils' level of knowledge and representations on health, illness, and the risk of disease is quite high. These studies allow us to understand how children think of health. They have a wide and varied notion of it. They can identify social and interpersonal determinants of health that coincide with their conceptions of health and their personal experiences of health (14). It would also appear that they are mindful of their health and have a positive attitude toward health promotion (16). The theme of nutrition is at the heart of children's perceptions, who attribute it a central role for being in good health (19, 23, 25). They often find their ideas through different categories of learning: self-reflective experience, experience of close contacts, casually watching others, and common discourse (15). Adolescents may also use different social network websites on which they share useful and interesting information on their health (18). The internet therefore appears as a tool for learning, communication, entertainment, and advice on health care (21). Through their productions, we also note that children acquire new conceptions of health and diseases. They can modify and expand them thanks to health education based on actions, with active and participative approaches to teaching and learning (22). Of course, the conceptions also vary with age. Older children have more varied conceptions of illness and health than younger ones. Illness is mainly perceived by them as a biomedical phenomenon and health as a psychosocial phenomenon (17, 24, 26). According to certain authors, there is therefore a solid basis for considering pupils in schools as “agents of health” (20). Within these studies, children and adolescents spoke about health with references to many health determinants except for a few that were not mentioned. These include factors linked to living and work conditions, factors linked to individual, biological, and genetic characteristics, and health inequalities (social, geographical, or sexual disparities).

Otherwise, the articles in this literature review have highlighted certain limits of the sampling and methodologies used (59).

The “draw and write” technique is a visual research method founded on art and developed for studies of child health in the 1980's in the United Kingdom. It allows us to assess the extent of children's understanding of health and the behaviors that are associated with it (19). The underlying fields of health are often conceptual and confusing. It is common to assume that young children know very little about health or that what they do know is wrong. This method provides a better understanding of the meaning they place behind “health,” “illness,” or “risk” (60, 61). The benefits of this method are numerous: the ease of collecting data, the fun and enjoyable aspect of its implementation for children, and the richness of the visual data. “The drawings offer another insight into the creation of human meaning besides written or spoken words, because they can express what is difficult to express: the ineffable, elusive, not-yet thought” (62). Nevertheless, there are certain limits, notably in terms of its validity and whether the drawing really expresses what they are meant to express (24). Children may draw what is easy to describe or can be affected by the other children or may even want to please the researcher. There may be a tendency to draw what they think is the “right answer” (63), choosing elements that do not really correspond to their ideas or opinions. Children think that they must give a correct answer, especially when they are in a school environment which therefore has a normative influence. The main problem linked to this technique is the difficulty in analyzing and interpreting, even overinterpreting, the resulting visual data, which can take a lot of time (64). Another limitation that is often identified is the use of a uniquely qualitative treatment without an analysis of the quantitative data (16).

It should also be noted that the study “Children's perspective of health and illness: images and lay concepts in preadolescence” (16) was within a specific cultural context: the results may not be transferable to other countries or other contexts. Consequently, additional comparative work must be done on this particular question.

The study on health literacy “The Relationship Between Health Literacy and Health Conceptualizations: An Exploratory Study of Elementary School-Aged Children” (14) was carried out in a single school, therefore representing a single geographical region. As such, the magnitude of children's experiences cannot be captured in this limited context for data collection. Moreover, the data may have been different if the researcher was not known by the children. At that young age, they can become anxious talking about health with someone they do not know.

The Spanish study “Children as agents of their own health: exploratory analysis of child discourse in Spain” also underlined sampling limits: the discussion groups were not statistically representative due to their sample size. As a result, the external validity of any conclusions cannot be guaranteed. Since school systems throughout Spain present similar organizational schemes (study programs, training of teaching staff, teaching methods, evaluation system, legal framework, and mixed schools), the reproduction of this study in other regions of the country would allow the rapid saturation of data or of similar results (20).

On the other hand, this study highlights certain obstacles to the use of focus groups. Leading discussion groups in a school environment during class and with pupils who already know each other may have an influence on the dynamics of the group.

In addition, the phases of data collection in focus groups make room for dialogue and exchanges to observe which elements are addressed by pupils in front of their peers. Focus groups also often serve to test or generate new unexpected ideas for the researcher and can highlight the argumentative lines developed by the children. However, it is important to remain prudent during interpretation, as focus groups essentially provide an array of points of view and opinions. The results cannot be applied to the community. The qualitative data are therefore difficult to interpret and analyze (21). Another point to remember is that, if the moderator is not well-trained, there is a risk of steering the responses. The limitations of focus groups also result from a blocking aspect of discussion groups: shyness or reticence (20). Certain subjects are difficult to address in front of a group and can halt or sometimes even modify the dialogue, thus generating bias in the data. In a group setting, participants have the tendency to lean on ideas that are considered correct from a socio-cultural point of view. Focus groups are a way of gathering the opinions of several people at the same time and therefore to benefit from the dynamics of a group but they can be more difficult to organize and set up than individual interviews.

Interviews facilitate the establishment of a relationship of trust and ease the comprehension of questions because the child can be given clarifications for misunderstandings, thus allowing the collection of more detailed data. One of the limits of interviews is the phenomenon of “social desirability” (65) which leads to the risk of children giving responses they think are adequate based on the expectations of the researcher, to ensure that the latter has a positive image of the former. Taking this risk into account, the data should be triangulated, which means it should be combined with other types of data collection to ensure the credibility of the data obtained. Furthermore, it is particularly hard to question children for several hours because they can tire of the classical interview situation and express discomfort or impatience (14, 26).

Photoelicitation allows, based on a corpus of images, the investigation of children's conceptions of health. This method, widespread in the United States in the field of marketing for example, is only cited in one of the selected international publications. However, the use of photographs is common in the field of prevention and is known to be effective and pertinent in research involving children (66) to explore their conceptions on matters of health: photovoice, photolanguage, photoexpression. This medium favors dialogue and enriches interactions (67). For the same photograph, two, three, or four interpretations can emerge from children because each of them can generate a different meaning. The focus is on the richness and diversity of the words that emerge thanks to the photographs. The latter enable the collection of data that are qualitatively different to those from verbal interviews or pictures alone. Due to the particularity and complexity of certain subjects addressed at this age, it is important to allow children to express themselves using tools that are adapted and meaningful for their level of understanding. Moreover, the media dimension of images eases the spontaneous construction of meaning by addressing the theme of interest and not its personal situation. It plays a fundamental ethical role that avoids all invasive aspects by maintaining a distance with regards to the theme being questioned (67).

From a methodological point of view, it is interesting to combine the use of photographs in association with narration and then with a focus group. Confronting the words put forward in the first phase within a group seems apt for further developing the conceptions identified or to help make new ones emerge.

This set of methodological approaches makes way for a socio-constructivist learning process, the emancipation of the subjects, the valorization of children's words, and provides an ethical framework founded on a respect for the subject (67). However, they do not allow a precise highlighting of the conceptions of children and adolescents of the determinants of health.

## Conclusion

Although research on children and adolescents' conceptions of health is developing, their understanding and analysis constitute a relatively poorly-studied subject of research at the international level. As demonstrated in this literature review, it has only been studied in a few countries. Moreover, the impact factor of reviews in which these articles are published is, for the majority, low. Furthermore, the field of research remains rather heterogeneous and vast for most of these studies. This appears to be due to a lack of hesitancy when agglomerating wide age groups, with little coherency from a developmental point of view.

We also note that few links are made between conceptions; we witness a juxtaposition of elements collected from children and an inventory of conceptions without any research on the links between them to understand how things are interconnected. It is therefore fundamental not to limit ourselves to a simple census of conceptions but to seek to better understand how they interact with each other. The result of simply gathering the conceptions of children shows that individual determinants are over-represented (diet, physical activity…) in comparison to collective determinants that are rarely mentioned (environment, pollution…). Moreover, the studies presented here choose a variety of methodological approaches but without any real exhaustivity, giving the impression of being partial or unfinished, and the feeling of having been put together over time and when opportunities linked to the research themes arose. They certainly put forward approaches that are adapted to a young audience, paving the way for interactions and facilitating dialogue, but their single use utilization is insufficient for analysis and for understanding the conceptions of young people on matters of health.

Otherwise, it appears that one-off data gathering is insufficient for collecting rich data and it remains difficult to identify the links between the data. On the other hand, the methodological elements set up are often the same as those used for questioning adults, but these methods are not always adapted for children.

All the methods mentioned above are promising and this literature review demonstrates that there is no methodological holy grail or perfect approach, and that it is difficult to invent new ones. Nevertheless, it is possible to combine them. Creating a protocol with several phases thus appears to be a pertinent methodological process for collecting data from children. These multi-phase protocols would lead to a better understanding of the way in which children and adolescents' conceptions of health determinants interact, combine, and influence each other in a reciprocal manner. Combining different methodologies allows a longer timeframe for data collection and thus promotes the complementarity of data. It is necessary to establish data collection situations that make sense to children and adolescents in order to involve them and guarantee good quality results. A single intervention cannot engage children and adolescents in a thorough process of research on thoughts and expressions whereas several phases gathering several different tools constitutes a methodological protocol that favors this in-depth approach. Combining the use of photographs, writing, narration, and speech within a group guarantees the articulation of young people and a certain complementarity in the tools used, which enhances the conceptions used and allows a better understanding of how these interact with each other. The use of multiple methods therefore presents many advantages such as stimulating and maintaining the interest of children, and the opportunity for the “triangulation” of the collection methods. Children and adolescents often know more than they say. It is therefore necessary to have adapted methods for collecting information, taking into account their competences and their cognitive capacity to enable communication between researchers and children.

## Data Availability Statement

All datasets generated for this study are included in the article/supplementary material.

## Author Contributions

MD made a substantial contribution to the design of this literature review, in the acquisition of data, their interpretation and analysis as well as in the writing of the manuscript. FP and CG participated in the selection of the articles of this literary review. FP, LG, and CG contributed to the critical revision of the manuscript for intellectual reasons. PB's contribution consisted of a thorough proofreading of the manuscript, particularly in terms of presentation and form. FP and LG have agreed to report on all aspects of the work by ensuring that issues related to the accuracy or integrity of any part of the work are investigated and addressed, and appropriate resolution. FP has definitely approved the version to publish.

### Conflict of Interest

The authors declare that the research was conducted in the absence of any commercial or financial relationships that could be construed as a potential conflict of interest.
